# A Landscape of the Genomic Structure of *Cryptococcus neoformans* in Colombian Isolates

**DOI:** 10.3390/jof9020135

**Published:** 2023-01-18

**Authors:** Luz Helena Patiño, Marina Muñoz, Angie Lorena Ramírez, Nórida Vélez, Patricia Escandón, Claudia-Marcela Parra-Giraldo, Juan David Ramírez

**Affiliations:** 1 Centro de Investigaciones en Microbiología y Biotecnología-UR (CIMBIUR), Facultad de Ciencias Naturales, Universidad del Rosario, Bogotá 111321, Colombia; 2 Unidad de Proteómica y Micosis Humanas, Grupo de Investigación en Enfermedades Infecciosas, Departamento de Microbiología, Pontificia Universidad Javeriana, Bogotá 111321, Colombia; 3 Departamento de Microbiología y Parasitología, Facultad de Farmacia, Universidad Complutense de Madrid, 28001 Madrid, Spain; 4 Grupo de Microbiología, Instituto Nacional de Salud, Bogotá 111321, Colombia; 5 Molecular Microbiology Laboratory, Department of Pathology, Molecular and Cell-Based Medicine, Icahn School of Medicine at Mount Sinai, New York, NY 10029, USA

**Keywords:** whole genome sequencing, *Cryptococcus neoformans*, SNP, CNV

## Abstract

*Cryptococcus neoformans* species complexes are recognized as environmental fungi responsible for lethal meningoencephalitis in immunocompromised individuals. Despite the vast knowledge about the epidemiology and genetic diversity of this fungus in different regions of the world, more studies are necessary to comprehend the genomic profiles across South America, including Colombia, considered to be the second country with the highest number of Cryptococcosis. Here, we sequenced and analyzed the genomic architecture of 29 Colombian *C. neoformans* isolates and evaluated the phylogenetic relationship of these strains with publicly available *C. neoformans* genomes. The phylogenomic analysis showed that 97% of the isolates belonged to the VNI molecular type and the presence of sub-lineages and sub-clades. We evidenced a karyotype without changes, a low number of genes with copy number variations, and a moderate number of single-nucleotide polymorphisms (SNPs). Additionally, a difference in the number of SNPs between the sub-lineages/sub-clades was observed; some were involved in crucial fungi biological processes. Our study demonstrated the intraspecific divergence of *C. neoformans* in Colombia. These findings provide evidence that Colombian *C. neoformans* isolates do not probably require significant structural changes as adaptation mechanisms to the host. To the best of our knowledge, this is the first study to report the whole genome sequence of Colombian *C. neoformans* isolates.

## 1. Introduction

*Cryptococcus neoformans* and *Cryptococcus gattii* species complexes are encapsulated obligated aerobic facultative intracellular fungal pathogens. These fungi, with *Candida* spp. and *Aspergillus* spp., are the most common etiological agents of opportunistic invasive fungal infections [1]. The *Cryptococcus neoformans* species complex predominantly affects immunocompromised patients, mainly HIV/AIDS individuals, with estimates of 223.100 new cases and more than 181.000 deaths globally per year. Sub-Saharan Africa has been considered to be the region with the highest incidence in cases of cryptococcal meningitis [2].

Although there are few reports regarding the incidence and prevalence of Cryptococcosis in Latin America, studies from Brazil and Colombia report an average annual incidence of 2.4 to 4.5 cases of meningeal Cryptococcosis per million inhabitants and a prevalence of 10 to 76% [3]. So far, Colombia is the second country in Latin America, after Brazil, with the highest number of cases reported of Cryptococcosis [4], with an average annual incidence of 2.4 cases per one million inhabitants and an incidence of 1.1 cases per 1000 people in HIV/AIDS patients [5].

Two species complexes of the genus *Cryptococcus* cause Cryptococcosis: *Cryptococcus neoformans* and *Cryptococcus gattii*. Several variants within the *Cryptococcus neoformans* species complex and *C. gattii* species complex have been recognized [6,7]. In the case of the *Cryptococcus neoformans* species complex, there are five main molecular types: VNI, VNII, VNB (collectively known as *C. neoformans*), VNIV (known as *C. deneoformans*), and VNIII (a hybrid of the two last varieties) [6,8]. In the *Cryptococcus gattii* species complex, there are six known molecular types: VGI (*C. gattii*), VGII (*C. deuterogattii* and subclassified into four genotypes: VGIIa, VGIIb, VGIIc, and VGIId [6,9]), VGIII (*C. bacillisporus*), VGIV (*C. tetragattii*), VGV, and VGVI (*C. decagattii*) [6,10]. Commonly, *Cryptococcus neoformans* var. *grubii* strains (VNI molecular type) are the most widely distributed all over the world. This type has been associated with 99% of infections in HIV patients with cryptococcal meningitis [6,11,12,13]. In Latin America, this molecular type is not only the principal etiologic agent of Cryptococcosis (~76%) but also the most recovered molecular type from environmental reservoirs (~69%) [3,5,14,15].

On the other hand, recent studies demonstrate how changes in the ecological niches caused by global warming and anthropomorphic factors have contributed to the expansion and survival of certain fungi shaping environmental pressures that result in emerging diseases. One of the fungi that have emerged to cause significant outbreaks and related to climate change is *C. gattii.* This fungus has migrated to territories with no previous reports; additionally, this species has increased the expression of certain virulence factors, favoring thus its pathogenicity and the emergence of infections in humans and animals [16,17].

Since the 1980s, different and efficient molecular typing methods have been developed; some of them used to study *Cryptococcus*’ epidemiology and genetic diversity [18,19,20,21,22]. So far, different molecular tools such as PCR-restriction fragment length polymorphism (RFLP), PCR fingerprinting, and Whole Genome Sequencing (WGS) have provided data for understanding the genetic structure of this species. Studies based in NGS have demonstrated not only the low genetic variability across *Cryptococcus* and the small number of chromosomal rearrangements between species and molecular types but also the variations in the gene content and the structural changes (aneuploidy, translocations, and inversions), mainly in *C. neoformans* [22]. Likewise, analyzing the population genetic structure within and between different lineages of *C. neoformans* species complexes identifies the microevolution signals of infecting strains [23], revealing the rapid capacity of adaptation of the organism through hypermutation [24] to identify genes related to the adaptation of stress conditions that include virulence and pathogenicity features [25,26,27], and the monitoring of outbreaks [28].

Additionally, WGS has revealed the presence of hybrids between the *Cryptococcus neoformans* species complex and *C. gattii* species complex [29]. These analyses are performed mainly with isolates from different geographical areas, such as North America, Asia, Africa, and Europe [25,30]. However, these hybrids still need to be discovered in other regions, such as South America. Although, Ashton et al. analyzed two isolates from Brazil and Argentina [30], and Firacative et al. analyzed two isolates of *C. gattii* from Colombia [31]. So far, no studies have analyzed the genomic diversity of *C. neoformans* in a representative number of samples.

Considering the high incidence of *C. neoformans* in Colombia [14,21] and the gap in the knowledge about the genomic features of this species in our country, this study focusses on analyzing the genomic characteristics of 29 *C. neoformans* Colombian clinical isolates recovered through the passive surveillance of Cryptococcosis led by the INS (National Institute of Health of Colombia). We evaluated not only the genomic variation among them (ploidy/gene changes and structural variations and single-nucleotide polymorphisms (SNPs)) but also their phylogenomic relationships with 582 available *C. neoformans* genomes, including genomes of different molecular types (VNI/VNII), hybrid genomes (*Cryptococcus neoformans* strain H99/*Cryptococcus neoformans* strain JEC21), and *Cryptococcus gattii* WM276 genomes.

## 2. Materials and Methods

### 2.1. Study Population

A total of 611 whole genomes of *Cryptococcus neoformans* species complexes were analyzed. These included 29 clinical isolates identified and confirmed by PCR fingerprinting and restriction-fragment length polymorphism (RFLP) as *C. neoformans* from the Microbiology Group of the INS, Bogotá, Colombia. The 582 genomes were downloaded from DDBJ/ENA/GenBank (http://www.ebi.ac.uk/ena). The clinical and epidemiological characteristics and MLST results (which followed the ISHAM MLST scheme consensus) of the 29 isolates here analyzed were previously described [21] and are presented in Table 1.

### 2.2. Whole Genome Sequencing

Each clinical isolate was cultured at 37 °C for 48 h in 2% Sabouraud Dextrose Agar (SDA) and later inoculated on yeast extract peptone dextrose agar (YEDP) at 37 °C for 24 h. One hundred microliters of each culture were harvested by centrifugation for a subsequent DNA extraction. This was conducted using the UltraClean^®^ Tissue and Cell DNA isolation Kit (MoBio Laboratories Inc., Carlsbad, CA, USA) as was described previously [21].

The extracted whole-genome DNA was sequenced using the Illumina HiSeq platform (Illumina; Novogene Bioinformatics Technology, Beijing, PR China). Mate-paired libraries were constructed by end repair (350 bp insert size) and subjected to paired-end sequencing (2 × 150 bp read length). The paired-end reads of each clinical isolate and the 611 global genomes (data downloaded from DDBJ/ENA/GenBank (http://www.ebi.ac.uk/ena, accessed on 10 January 2023) were mapped to the H99 reference genome of *C. neoformans* (RefSeq assembly accession GCF_000149245.1) and assembled with the smalt program (version 0.7.4) (http://www.sanger.ac.uk/science/tools/smalt-0, accessed on 10 January 2023). The mapping conditions involved the following parameters: an exhaustive search option (−x and −y 0.8), a reference hash index of 13 bases, and a sliding step of 3. An identity threshold of y = 0.8 prevented the mapping of non-*Cryptococcus* reads to the reference sequences. The read file merging, sorting, and elimination of PCR duplicates was implemented with SAMtools (version 0.1.18) and Picard (version 1.85) [32]

### 2.3. Phylogenomic Inferences

The phylogenomic relationships of 29 Colombian genomes sequenced in this study were evaluated globally by comparison against 582 publicly available genomes. Of the latter, 498 corresponded to the *C. neoformans* VNI molecular type, 56 to the *C. neoformans* VNII molecular type, 14 to the hybrids isolates of the *Cryptococcus neoformans* strain H99/*Cryptococcus neoformans* strain JEC21, and 14 corresponded to the isolates of *Cryptococcus gattii* WM276 [30]. Additionally, we included genomes representative for each of the four molecular types previously described for *C. neoformans*, as follows: ERR1395134 (VNI), ERR1395124 (VNII), ERR1520667 (VNIV), and ERR1520666 (VNB) [26]. The SNPs from the whole nuclear genome of each sample were extracted and used to build alignments. A maximum likelihood (ML) tree was then obtained in IQtree2 v.1.6.1 [33], using the best substitution model, default heuristic search options, and ultrafast bootstrapping with 1000 replicates and other parameters by default. The phylogenomic tree obtained was graphically represented in the Interactive Tree of Life online tool [34]. To identify closely related clusters as an indicator of the central *Cryptococcus* populations, a NeighbourNet network was reconstructed from the whole genome SNPs alignment in SplitsTree5 software v4.17.0 [35] to confirm the clusters generated by ML phylogeny. We included the *C. neoformans* strain H99 as the reference genome and *C. gattii* WM276 as the outgroup for all inferences.

### 2.4. Gene Copy Number Variations

To determine the chromosomal somy, the median read depth of each chromosome was estimated (di). Positions with a read depth of >1 standard deviation were removed, and the di was recalculated. Later, we calculated the median depth (dm) of the whole genome (14 chromosomes) and the somy (S-value) of each chromosome. The regions displaying a coverage equal to two were deemed as diploid events, and the ranges of triploid, tetraploid, and pentaploid events were defined as previously described [23]. To evaluate the gene copy number variations (CNVs), the average read depth of each copy number variation (CNV) call was calculated and compared with the average read depth of each chromosome [36]. The statistical significance used in this study was set at a z-score cutoff of >2 and an adjusted *p*-value (Student’s *t*-test) of <0.05. Gene Ontology was used for enrichment analyses from FungiDB tools (https://fungidb.org, accessed on 10 January 2023) and to evaluate the genes with CNVs. *p*-values were adjusted for multiple testing with the Benjamini–Hochberg method with a false-discovery rate (FDR) of <0.05. The GO terms were submitted to REVIGO for functional purposes [37].

### 2.5. Variant Prediction Calling, SNP Filtering, and Genetic Diversity Analysis

The reads were aligned to the closest reference genome available, the *C. neoformans* strain H99 [38], using the SMALT program (version 0.7.4). The merging and sorting of the bam files and marking duplicated reads were implemented with the Picard program (version 1.85) (http://broadinstitute.github.io/picard/, accessed on 10 January 2023) as described previously [39]. The SNPs were called using the Genome Analysis Toolkit (GATK) (version 3.4; https://software.broadinstitute.org/gatk/, accessed on 10 January 2023) Unified Genotyper with the haploid ploidy setting. We realigned around indels to remove these and retrieved only the SNPs. GATK Variant Filtration was used to filter low-quality SNPs according to the following criteria: QD < 2.0 || MQ < 40 || FS > 60.0 || ReadPosRankSum < −8.0. The SNP quality cutoff was set at 3000. The resulting variants were analyzed using SnpEff to determine the impact of SNPs [40], where SNPs with a high and moderate impact were selected. From this selection, we identified and analyzed the unique/shared SNPs between/within sub-lineages/sub-clades. Lastly, the total number of mutations and the nucleotide diversity was estimated using DNAsp v. 5.0. The Phidias bioinformatics resources https://www.phidias.us/victors/search_process.php (31 October 2022) and database of Virulence Factors in Fungal Pathogens http://sysbio.unl.edu/DFVF/GeneSearch-1.php (31 October 2022) were used to review the genes involved in the virulence of *C. neoformans.*

## 3. Results

### 3.1. Phylogenomic Analysis

**Figure 1:**
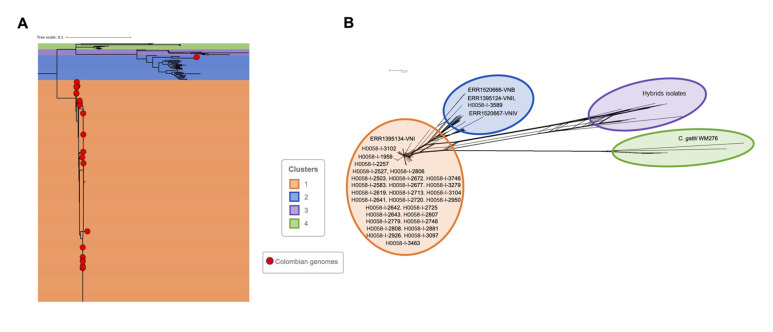
Phylogenetic relationship among the *Cryptococcus* genomes. (**A**) The figure represents the phylogenomic inference based on whole genome SNP alignments for the 611 genomic sequences analyzed in this study. The red dots represent the 29 Colombian genomes. (**B**) Phylogenetic networks constructed in SplitsTree 5, based on SNP alignments for 611 genomes analyzed. *C. neoformans* strain H99 was used as the reference genome, and *C. gattii* WM276 as an outgroup.

The topology of the ML tree using the complete dataset (*n* = 611) in IQtree2 revealed a grouping in four main clusters that represent the known *Cryptococcus* genomic population structure. Although the classification by the molecular type of publicly available genomes is limited to a small group of representatives, this information was enough to correlate the results with this traditional classification. The most abundant population was Cluster 1, which included 526 genomes corresponding to VNI. The other clusters had a lower abundance, reaching 57 genomes for Cluster 2, corresponding to VNII, 14 genomes for Cluster 3, corresponding to the hybrids isolates of the *Cryptococcus neoformans* strain H99/*Cryptococcus neoformans* strain JEC21 [30], and 14 genomes in cluster 4 corresponded to the isolates of *C. gattii* WM276, as previously published [30]. In the case of the Colombian genomes sequenced in this study, most of these were included in Cluster 1 (*n* = 28), and only one was included in Cluster 2 (H0058-I-3589) (Figure 1).

**Figure 2:**
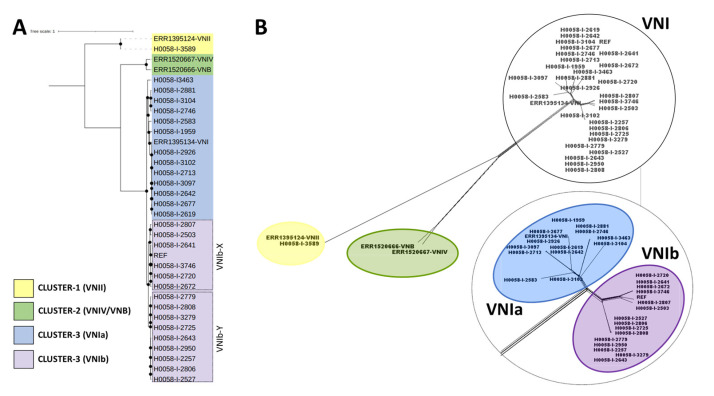
Phylogenetic analysis of Colombian *Cryptococcus neoformans* genomes. (**A**) Phylogenomic inference based on SNP alignments for the 29 Colombian genomic sequences included in this study. Black dots represent well-supported nodes (bootstrap ≥ 90). (**B**) Phylogenetic networks constructed in SplitsTree 5, based on SNP alignments for 29 Colombian genomes. The bottom figure represents the magnification of cluster 3 (VNI) to visualize the VNI sub-lineages. Genomes representatives for each of the four molecular types previously described for *C. neoformans* were included: VNI (ERR1395134), VNII (ERR1395124), VNIV (ERR1520667), and VNB (ERR1520666).

A detailed analysis based on SNPs was conducted to evaluate the phylogenetic relationships among the 29 samples analyzed in this study and the reference genomes of *C. neoformans* molecular types. We observed three clusters in well-supported nodes. Cluster 1 (VNII) included the reference genome representative of VNII (ERR1395124) and the H0058-I-3589 genome. Cluster 2 included the reference genomes representative of VNIV and VNB (ERR1520667 and ERR1520666, respectively), and Cluster 3 (VNI), which included 28 of the 29 genomes analyzed in this study, the reference strain *C. neoformans* strain H99 and the VNI (ERR1395134) (Figure 2). In addition, the tree topology showed two sub-lineages in cluster 3: VNIa and VNIb (represented by 13 and 15 isolates, respectively) and two sub-clades within VNIb sub-lineage in well-supported nodes, called VNIb-X and VNIb-Y. This cluster with VNIb-X accounts for 40% of the genomes (some close to the *C. neoformans* strain H99 reference genome) and VNIb-Y for 60% (Figure 2A). These findings were additionally supported by the phylogenetic tree topologies obtained in SplitsTree5 (Figure 2B) where the number and distribution of the clades were consistently clustered together. Finally, we did not observe relationships between the clustering patterns with the clinical and demographic characteristics of each isolate analyzed.

### 3.2. Gene Copy Number Variation Analysis (CNV)

Initially, we estimated and compared the copy numbers per chromosome in all genomes analyzed. The results showed a whole karyotype, with no evidence of aneuploidy in any 14 chromosomes. Subsequently, we evaluated and compared the genes that presented CNVs (z-score cutoff > two and adjusted *p*-value < 0.05) in each clinical isolate. The results showed moderate differences among them; the genome with the highest number of genes with CNV was H0058-I-2881 (125 genes), and the lowest was H0058-I-2641 (51 genes). The Gene Ontology enrichment analysis of the genes that presented CNVs in each isolate revealed that the terms with the highest enrichment for biological processes were associated with the cellular metabolic and catabolic processes. The terms for the molecular processes were associated with the oxidoreductase and hydrolase activity. For cellular processes, the terms with the highest enrichment were associated with the intracellular membrane and rough endoplasmic reticulum (Figure S1).

**Figure 3:**
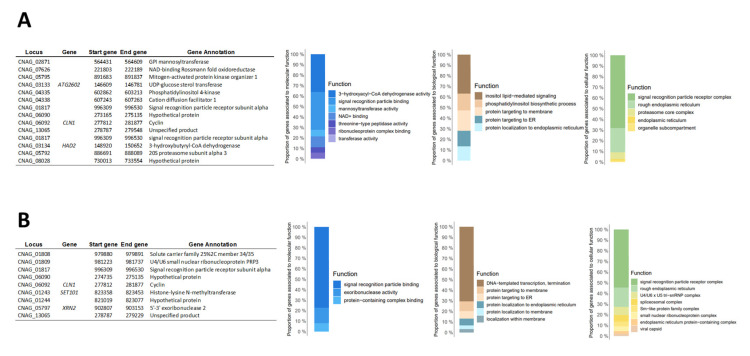
Evaluation of genes with copy number variation (CNV) across VNIa and VNIb sub-lineages. The table shows the genes with CNV shared between the genomes belonging to VNIa (**A**) and VNIb (**B**). On the right, the Gene Ontology enrichment analysis to functional categories (molecular, biological, and cellular) for each sub-lineage (VNIa and VNIb).

Lastly, we grouped the isolates belonging to VNI, according to the sub-lineages (VNIa and VNIb), excluding the unique isolate belonging to VNII (H0058-I-3589). We identified the genes with CNV shared within them (we averaged the values obtained in each genome and calculated the standard deviations σ and the corresponding z-score). The results evidenced a low number of genes with CNV shared between each sub-lineage. Fourteen to VNIa and nine to VNIb, highlighting in VNIa the genes CNAG_02871, CNAG_03133 (ATG2602), CNAG_04335, CNAG_06092 (CLN1), and CNAG_03134 (HAD2) (Figure 3A), and in VNIb, the genes CNAG_01809, CNAG_06092 (CLN1), CNAG_01243 (SET101), and CNAG_05797 (XRN2) (Figure 3B), associated with the critical process of survival and virulence in the fungi.

**Figure 4:**
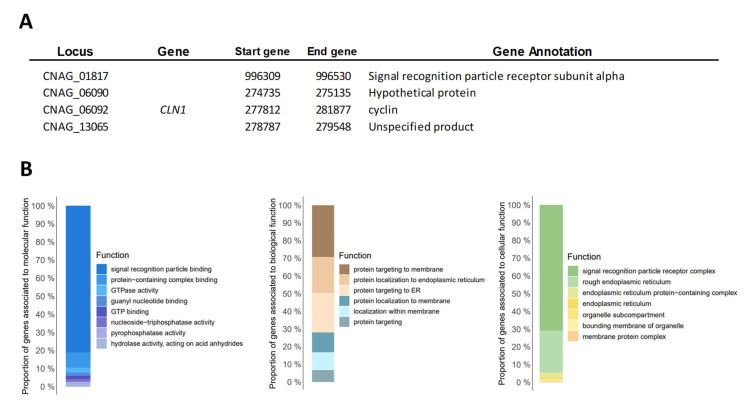
Evaluation of genes with copy number variation (CNV) shared between the 29 Colombian *Cryptococcus neoformans* genomes. (**A**) The genes with CNV were shared between the 29 isolates included in the study. (**B**) Gene Ontology enrichment analysis to functional categories (molecular, biological, and cellular).

The Gene Ontology enrichment analysis to functional categories for the sub-lineages of the VNI molecular type is shown in Figure 3. Finally, we identified the genes with shared CNV between the 29 genomes analyzed independently of the molecular type. Four genes with shared CNV were observed (CNAG_01817, CNAG_06090, CNAG_06092, and CNAG_13065); two of them were annotated with known functions (Signal recognition particle receptor subunit alpha and cyclin) and two as hypothetical proteins (Figure 4A). Additionally, we identified whether functional categories (molecular, biological, and cellular in Gene Ontology) were enriched among the genes shared. The terms “Signal recognition particle binding” to molecular processes, the terms “Protein targeting to membrane” to biological processes, and the terms “Signal recognition particle receptor complex” to cellular processes were the terms that presented more enrichments (Figure 4B).

### 3.3. Single Nucleotide Polymorphisms Analysis

**Figure 5:**
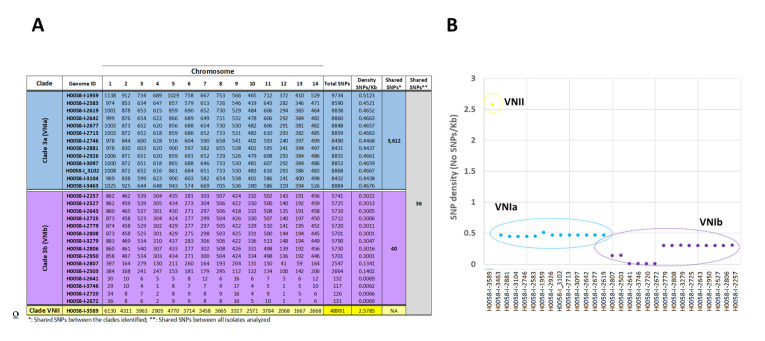
The landscape of SNPs identified in the 29 Colombian *Cryptococcus neoformans* genomes. (**A**) The figure describes the number of SNPs identified per chromosome, with a potential effect on gene function, in each genome analyzed, as well as the number of shared SNPs in the VNI sub-lineage (VNIa and VNIb) and the number of shared SNPs between the 29 genomes included in the study. (**B**) The density of SNPs in each of the 29 genomes was analyzed.

A total of 321,268 SNPs were identified by comparing each of the 29 clinical isolates with the reference genome *C. neoformans* strain H99. Around the 75% of variants observed presented a low and modifier impact and between 21 and 25% potentially affected the gene function (a high and moderate impact) (Table S1). The isolates with the lowest and highest number of SNPs with a functional impact were H0058-I-3746 (117 SNPs) and H0058-I-1959 (9734 SNPs) isolates, respectively (Figure 5A). To analyze the number of potential effects of the SNPs between each molecular type, we observed 15,674 SNPs for VNI and 48,991 for VNII; only one isolate fell in the VNII molecular type (H0058-I-3689) and the increase in the number of SNPs. This could be explained by the fact that this isolate was mapped to a reference genome belonging to a different molecular type (VNIb); we decided to exclude this sample from further analysis and focus on the isolates belonging to the VNI molecular type and its divergent sub-lineages/sub-clades (VNIa and VNIb (VNIb-X and VNIb-Y)).

Initially, we evaluated the genetic variability of Colombian *C. neoformans* VNI isolates and compared them with the genetic variability reported for VNI from Asia and Africa. The results evidenced a comparable genetic variability between Colombian and African isolates (102559 SNPs (*n* = 29) and 105391 SNPs (*n* = 24), respectively) and a low variability in the Asian isolates (~48,000 SNPs; *n* = 50).

Lastly, we evaluated the total number of SNPs with a potential effect per chromosome in each isolate. The results showed for the VNIa sub-lineage a homogeneous distribution of SNPs across the isolates studied (~8000 to 10,000 SNPs), H0058-I-2881 and H0058-I-1959 being the genomes with the lowest and highest number of SNPs (8431 and 9734, respectively). In contrast with the VNIb sub-lineage, where 11/15 isolates presented several SNPs ranging from ~2000 to 5700 SNPs, and in 4 isolates, some SNPs ranging from 117 to 132; H0058-I-3746 and H0058-I-3279 being those isolates with the lowest and highest number of SNPs (117 and 5.790, respectively) (Figure 5A). Regarding the total number of variants per chromosome, an association between the number of SNPs and the chromosome size was observed; the larger chromosomes (chromosomes 1, 5, and 11) presented a more significant number of SNPs than shorter chromosomes (chromosomes 12 and 13) (Figure 5A).

Subsequently, we analyze the shared SNPs (with a high and moderate functional impact) within sub-lineages/sub-clades belonging to the VNI molecular type. The comparison of genomes within this group revealed that the isolates of the VNIa sub-lineage had the highest number of shared SNPs (3612) (21 of them with a high functional impact: e.g., stop_gained and stop_lost) (Table S2; Figure 5A), which contrasted with the low number of shared SNPs observed in the VNIb sub-lineage (40 SNPs). Considering the sub-clades identified within the VNIb sub-lineage, we compared them; the results showed 67 shared SNPs between the isolates belonging to the VNIb-X sub-clade and 5542 SNPs in the VNIb-Y sub-clade.

Lastly, we evaluated the shared SNPs between the 28 genomes analyzed. A total of 36 shared SNPs with a moderate functional impact, 41% in genes encoding proteins associated with hypothetical function, and 59% in genes with a known function were observed. Such as transporter proteins (sodium-independent sulfate anion transporter and ferric transporter), proteins associated with the metabolism (alpha-mannosidase, oligosaccharyltransferase, and acetolactate synthase 2C small subunit), and proteins associated with the virulence (urate oxidase, CAMK/CAMKL/AMPK protein kinase, antiviral helicase SKI2) (Table S3).

Finally, we evaluated the SNP density in each clinical isolate and the nucleotide diversity (π) in the different sub-lineages. We found a moderate polymorphism in the isolates belonging to the VNIa sub-lineage, with an SNP density/Kb between 0.4 and 0.5 and a value of π = 0.061 and a low variability within the isolates belonging to the VNIb sub-lineage with an SNP density/Kb between 0.006 and 0.3 and a value π = 0.042. Interestingly, we observed that the isolates belonging the VNIb-X sub-clade presented the lowest variability (ranging between 0.13 and 0.006 SNPs/Kb) compared with the VNIb-Y sub-clade, which evidenced a variability of ~0.3 SNPs/Kb. These results contrasted with the H0058-I-3589 isolate belonging VNII sub-lineage with a high polymorphism of 2.5 SNPs/Kb (Figure 5B).

## 4. Discussion

Cryptococcal meningitis infections are considered one of the principal causes of mortality globally in AIDS-related deaths. *Cryptococcus neoformans* is the most widely distributed all over the world. [38]. Considering that this species has been the most frequently recovered from clinical isolates [5] and with a wide distribution in different departments and Colombian environments [14,15], this study focused on analyzing the phylogenomic relationship and genetic structure of *C. neoformans* from 29 Colombian clinical isolates of cerebrospinal fluid.

The phylogenetic analysis performed between the 611 genomes included in the study showed that the vast majority of isolates (97%) corresponded to the VNI molecular type (Figure 1 and Figure 2A), confirming previous studies performed by MLST [5,14] and its global distribution [41,42]. By analyzing the genetic variability of VNI and comparing it with the reported isolates from Africa and Asia, the *C. neoformans* VNI lineage from Colombia presents a comparable variability with isolates from Africa [43] (Colombian isolates = 102,559 SNPs (*n* = 29) vs. African isolates = 105,391 SNPs (*n* = 24)). This includes a high variability compared with Asian isolates ((~48,000 SNPs; *n* = 50) [30], which could suggest possible migration events between isolates from South America and Africa, as has been proposed for the VNB lineage [44]. Nevertheless, an additional analysis with South American VNI isolates is necessary to determine this premise.

The phylogeny strongly supported two of the three sub-lineages of the *C. neoformans* VNI lineage identified so far (VNIa and VNIb) (Figure 2). These results are consistent with the SNPs analysis and nucleotide diversity observed in each sub-lineage. A total of 8.800 SNPs (3612 shared) with a density of 0.4 SNPs/Kb and a value π = 0.061 within the VNIa sub-lineage and 3.800 SNPs (40 shared) with a density of 0.2 SNPs/Kb and a value π = 0.042 within VNIb were identified (Figure 5), thus revealing the intraspecific divergence of VNI, as has been previously reported in isolates from different regions of the world [25,30]. To analyze the diversity within each sub-lineage more deeply, we did not observe diversification events within the VNIa sub-lineage. However, in the VNIb sub-lineage, we could observe two well-supported groups (VNIb-Y and VNIb-X) (Figure 2), one of them being six times more diverse than the other (5752 vs. 952 SNPs) (0.30 vs. 0.05 SNPs/Kb). So far, three distinct sub-clades within the *C. neoformans* VNIa sub-lineage (VNIa-4, VNIa-5, and VNIa-93) [30] have been identified. However, no sub-clades within the *C. neoformans* VNIb sub-lineage have been previously identified from any global collection. Therefore, this is the first study that reports diversification events within the VNIb sub-lineage.

Overall, these findings describe (i) the genomic structure of *Cryptococcus neoformans* in Colombian isolates, which are predominantly clonal and correspond primarily to VNIa, as previously described in South America [45] and from different regions of the world [30,46]. (ii) The elevated number of changes in the VNIa sub-lineage is possibly explained by adaptation changes, as has been described for other lineages of *C. neoformans* and *C. gatti* [8] and other fungi such as *Candida albicans* [47,48], or probably by the use of a reference genome belonging to a different sub-lineage (VNIb). (iii) The diversification within the VNIb sub-lineage, which together with its low diversity and the presence of unique SNPs, could suggest an early diversification process or even an effective adaptation in the host. This might be favoring specific genotypes, as observed in some *Cryptococcus* species, where structural changes might participate in the adaptative diversification and to contribute to the assimilation of specific metabolites [49], or the expansion of the telomere-associated (TLO) genes family and the variations in the DNA binding of the Sterol Regulatory Element Binding Proteins (SREBPs) [50,51,52]. Despite the results obtained, additional studies with a deeper sampling from South America (including Colombia) must confirm these findings in order to identify the possible presence of other molecular types and to establish how these sub-lineages are diversifying and expanding in and out of the continent.

Subsequently, we analyzed the structural changes at the chromosome/gene level. Accumulating evidence indicates that aneuploidy could occur commonly in fungi and other eukaryotic cells spontaneously or because of genetic or environmental perturbations [53]. However, this mechanism has been widely observed in *C. neoformans* species complexes, mainly in drug-resistant populations [54] or under growth conditions [55]. In this study, we identified homogeneity in the somy in the Colombian *C. neoformans* isolates, which could be explained as a possible adaptation of fungi to culture or due to the low pressure associated with an antifungal treatment (all isolates analyzed were sensible to fluconazole treatment) [21]. This low structural variation observed in *C. neoformans* VNI was reinforced by the low number of genes with CNVs between and within VNIa and VNIb sub-lineages. Despite these findings, we highlight variations in the genes previously identified by other authors [56,57] and associated directly with the growth, cell wall integrity, transcription, cell cycle, metabolism, and virulence of *C. neoformans* species complexes (GPI mannosyltransferase, mitogen-activated protein kinase organizer 1, UDP-glucose sterol transferase, U4/U6 small nuclear ribonucleoprotein PRP3, Phosphatidylinositol 4-kinase, Cyclin, Histone-lysine N-methyltransferase, and 5’-3’ exoribonuclease 2) [58,59,60] (Figure 3). These findings suggest that the Colombian *C. neoformans* VNI isolates favor changes in specific genes essential to survival, proliferation, and virulence within the host (phenotypic switching) as a possible adaptation mechanism, as has been previously described in other fungi species [61,62,63]. Additional studies with mutant strains could contribute not only to confirming this hypothesis but also to expanding the knowledge associated with the life cycle of this fungus.

Finally, to obtain more information about the genomic variability in the Colombian *C. neoformans* VNI isolates, we analyzed in detail the nucleotide-level variation (SNPs). However, we did not observe significant structural changes within and between the VNIa and VNIb sub-lineages. We observed a marked difference in the number of SNPs (3260 and 40 SNPs, respectively). Interestingly, some of the SNPs found in the VNIa sub-lineage introduced premature stop/start codon in genes associated with the intracellular trafficking from ER to the Golgi apparatus (CNAG_00064) [64], with the metabolism and signal pathways (CNAG_002551 and CNAG_02920) or with the transcription (CNAG_07689) (Table S2). Additionally, to analyze the shared SNPs between all *C. neoformans* VNI isolates, we observed SNPs in the genes involved in the essential biological pathways of the fungi, such as transport, the metabolism, and signal pathways, and mainly in virulence, highlighting the CFT1 gene required for the fungi to use host iron sources and to cause disease. This deletion has been demonstrated to attenuate the virulence in an animal model [65], the PKA2 gene which regulates the mating, haploid fruiting, and virulence in serotype D strains of *Cryptococcus* [66]. The URO1 gene, known as dispensable for virulence [67], and the SKI2 gene, whose mutant exhibited a lower virulence and susceptibility to anti-ribosomal drugs [68] (Table S3). These results describe that *C. neoformans* VNI present changes in specific genes that could not only favor its survival and adaptation but also possibly modify the clinical and therapeutic outcomes.

We recognize that this study presents some limitations, such as the small number of samples analyzed; hence, its underrepresentation only allows us to describe the diversity in some Colombian territories. Additionally, the sampling was performed at a unique moment. We consider that is necessary to analyze the genomic architecture of Colombian *C. neoformans* isolates under certain stress conditions (nutritional, environmental, or in the presence of some azole drugs) to see if these types of stresses can impact the ploidy, SNPs, or CNVs across the genomes.

## 5. Conclusions

In conclusion, this is the first study to report the genome sequencing of Colombian *C. neoformans* isolates. We observed the high genetic variability of this species (VNI being the dominant genotype) and the presence of two sub-lineages (VNIa and VNIb) and two sub-clades within the VNIb sub-lineage which were well-supported, demonstrating the intraspecific divergence. Likewise, we observed that minimal changes in terms of the SNPs and gene CNV levels could be used for *C. neoformans* as possible mechanisms of an adaptation to the host. Further sampling and additional analysis are required to confirm these hypotheses.

## Figures and Tables

**Table 1:** Clinical and demographic variables and MLST and WGS results of 29 clinical isolates of *C. neoformans*.

Isolate ID	Age	Sex	Department	AIDS	ST	RFLP	Cluster (WGS)
H0058-I-3589	44	Male	Cundinamarca	Negative	100	VNII	Cluster-1 (VNII)
H0058-I-2583	37	Male	Antioquia	Positive	377	VNI	Cluster-3 (VNIa)
H0058-I-2619	43	Male	Antioquia	Positive	377	VNI	Cluster-3 (VNIa)
H0058-I-2642	48	Female	Antioquia	Positive	377	VNI	Cluster-3 (VNIa)
H0058-I-2746	34	Female	Valle	Positive	5	VNI	Cluster-3 (VNIa)
H0058-I-2881	68	Female	Valle	Negative	298	VNI	Cluster-3 (VNIa)
H0058-I-3097	21	Female	Antioquia	Positive	95	VNI	Cluster-3 (VNIa)
H0058-I-3102	31	Male	Antioquia	Positive	377	VNI	Cluster-3 (VNIa)
H0058-I-3104	48	Female	Meta	Positive	5	VNI	Cluster-3 (VNIa)
H0058-I-3463	31	Male	Atlantico	Positive	6	VNI	Cluster-3 (VNIa)
H0058-I-1959	54	Male	N. santander	Negative	77	VNI	Cluster-3 (VNIa)
H0058-I-2677	72	Male	Antioquia	Positive	95	VNI	Cluster-3 (VNIa)
H0058-I-2713	ND	ND	ND	ND	95	VNI	Cluster-3 (VNIa)
H0058-I-2926	ND	ND	ND	ND	95	VNI	Cluster-3 (VNIa)
H0058-I-2503	33	Male	N. Santander	Positive	63	VNI	Cluster-3 (VNIb-X)
H0058-I-2807	30	Male	Antioquia	Positive	23	VNI	Cluster-3 (VNIb-X)
H0058-I-3746	48	Male	Cauca	Negative	2	VNI	Cluster-3 (VNIb-X)
H0058-I-2641	ND	ND	ND	ND	2	VNI	Cluster-3 (VNIb-X)
H0058-I-2672	ND	ND	ND	ND	2	VNI	Cluster-3 (VNIb-X)
H0058-I-2720	ND	ND	ND	ND	2	VNI	Cluster-3 (VNIb-X)
H0058-I-2257	26	Male	Antioquia	Positive	69	VNI	Cluster-3 (VNIb-Y)
H0058-I-2527	ND	ND	ND	ND	69	VNI	Cluster-3 (VNIb-Y)
H0058-I-2643	46	Male	Antioquia	Positive	69	VNI	Cluster-3 (VNIb-Y)
H0058-I-2725	39	Male	Antioquia	Positive	69	VNI	Cluster-3 (VNIb-Y)
H0058-I-2779	39	Male	Antioquia	Positive	69	VNI	Cluster-3 (VNIb-Y)
H0058-I-2806	36	Male	Antioquia	Positive	69	VNI	Cluster-3 (VNIb-Y)
H0058-I-2808	31	Male	Antioquia	Positive	69	VNI	Cluster-3 (VNIb-Y)
H0058-I-2950	44	Male	Valle	Positive	69	VNI	Cluster-3 (VNIb-Y)
H0058-I-3279	ND	ND	ND	ND	69	VNI	Cluster-3 (VNIb-Y)

AIDS: acquired immunodeficiency syndrome. ST: sequence type. RFLP: restriction fragment length polymorphism of the URA5 gene. Cluster (WGS): cluster identified by whole genome sequencing. ND: no data.

## Data Availability

All experimental data are provided in the manuscript and in supplementary files or are available via the NCBI BioProject database, with the accession number PRJNA669191 previously reported [18].

## Supplementary Materials

The following supporting information can be downloaded at: https://www.mdpi.com/article/10.3390/jof9020135/s1, Figure S1. Genes with copy number variation (CNV) in each of the 29 Colombian *Cryptococcus neoformans* genomes; Table S1: Number of SNPs with synonymous variant effect or relevant impact on gene function. Table S2: List of SNPs with high functional impact identified between the 13 isolates belong to VNIa sub-lineage; Table S3: List of shared SNPs between the 29 isolates analyzed in this study.
