# Supplementary figures and images for: A Landscape of the Genomic Structure of Cryptococcus neoformans in Colombian Isolates
Source: J Fungi (Basel). 2023 Jan 18;9(2):135. doi: 10.3390/jof9020135 (PMC9959405; doi:10.3390/jof9020135)

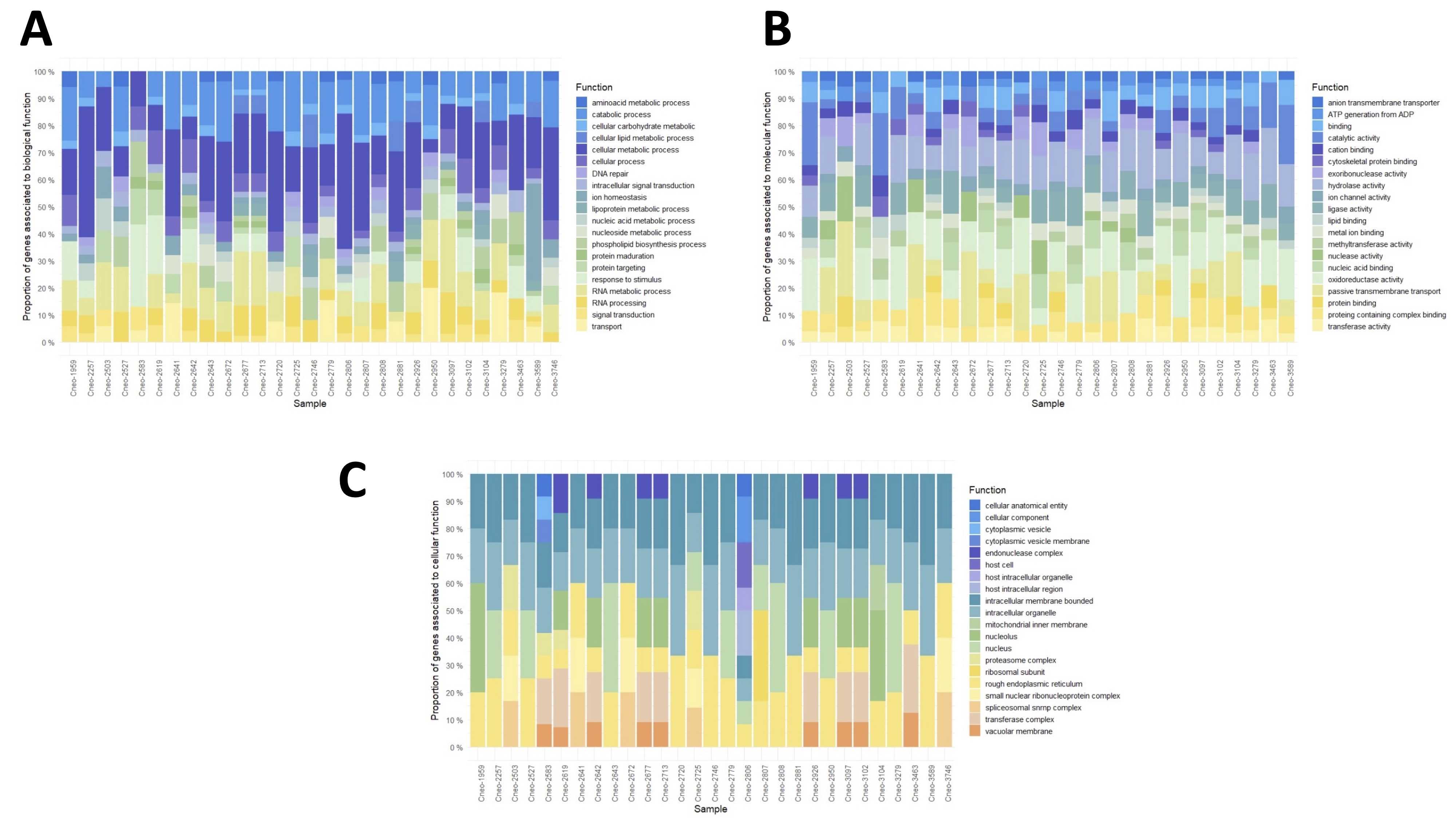

Supplement: Supplementary file 1 [file jof-09-00135-s001.zip › Supplemental material/Figure S1.jpeg]
